# Breed-specific meat quality and flavor characteristics of Wannan and Chinese Simmental cattle revealed by whole transcriptome and metabolome analysis

**DOI:** 10.1016/j.fochms.2026.100451

**Published:** 2026-09-01

**Authors:** Ning Song, Jia Wang, Xiang Meng, Shuangshuang Cui, Sihua Jin, Hang Tang, Yunhai Zhang, Hongyu Liu

**Affiliations:** a Anhui Province Key Laboratory of Local Livestock and Poultry, Genetical Resource Conservation and Germplasm Innovation, College of Animal Science and Technology, Anhui Agricultural University, Hefei 230036, China; b Institute of Beef Cattle Industry, Anhui Agricultural University, Hefei 230036, China

**Keywords:** Beef, Amino acids, Fatty acids, Whole transcriptomics, Metabolomics

## Abstract

Deciphering the genetic and metabolic factors influencing meat quality and flavor is crucial for enhancing beef industry productivity and meeting consumer demands. This study aimed to explore the effects of different breeds on meat quality and flavor characteristics of Wannan and Chinese Simmental cattle through analysis of myofiber traits, fatty acid and amino acid contents, whole transcriptomics, and metabolomics. Results revealed that Wannan cattle had markedly smaller myofiber diameter (*P* < 0.01) and higher myofiber density (*P* < 0.05) than Chinese Simmental cattle. Wannan cattle also exhibited significantly higher contents of umami−/sweet-related amino acids (aspartic acid and glycine) (*P* < 0.05), and unsaturated fatty acids (C15:1, C16:1, and C18:2n6c) (*P* < 0.05), as well as higher levels of total unsaturated (UFAs), monounsaturated (MUFAs), and polyunsaturated fatty acids (PUFAs) (*P* < 0.05). We identified key genes involved in fatty acid metabolism (*FADS6*, *PLB1*, *ELOVL6*, *HACD2*) and amino acid metabolism (*ADSS2*, *GSTA2*, *SLC36A4*), as well as core signaling pathways including PI3K-Akt, AMPK, and PPAR. Additionally, lncRNA/circRNA-mediated potential competing endogenous RNA regulatory networks were constructed, represented by LOC112449549/miR-424/ELOVL6, circ_015602/miR-2285/HACD2, and circ_035929/miR-194-3p/SLC36A4 axes. Further, key metabolites in fatty acid metabolism (16:0 PC, 16:1 (Δ9-Cis) PC, 18:1 Dimethyl PE) and amino acid metabolism (gamma-Glu-Cys, 1-methyl-L-histidine, l-glutamine) were identified, and an mRNA-metabolite regulatory network was established. The findings are expected to contribute to the genetic improvement of local cattle breeds and promote the development of high-quality beef production in China.

## Highlights

• Wannan cattle exhibits smaller myofiber diameter and higher myofiber density.
• Wannan cattle contains higher content of aspartic acid, glycine, C15:1, C16:1, C18:2n6c.
• Fatty acid composition was influenced by *FADS6*, *PLB1*, 16:0 PC, 16:1 (Δ9-Cis) PC, 18:1 Dimethyl PE.
• Amino acid composition was influenced by *GSTA2*, *ADSS2*, γ-Glu-Cys, 1-methyl-L-histidine, l-glutamine.

## Graphical abstract

**Figure:**
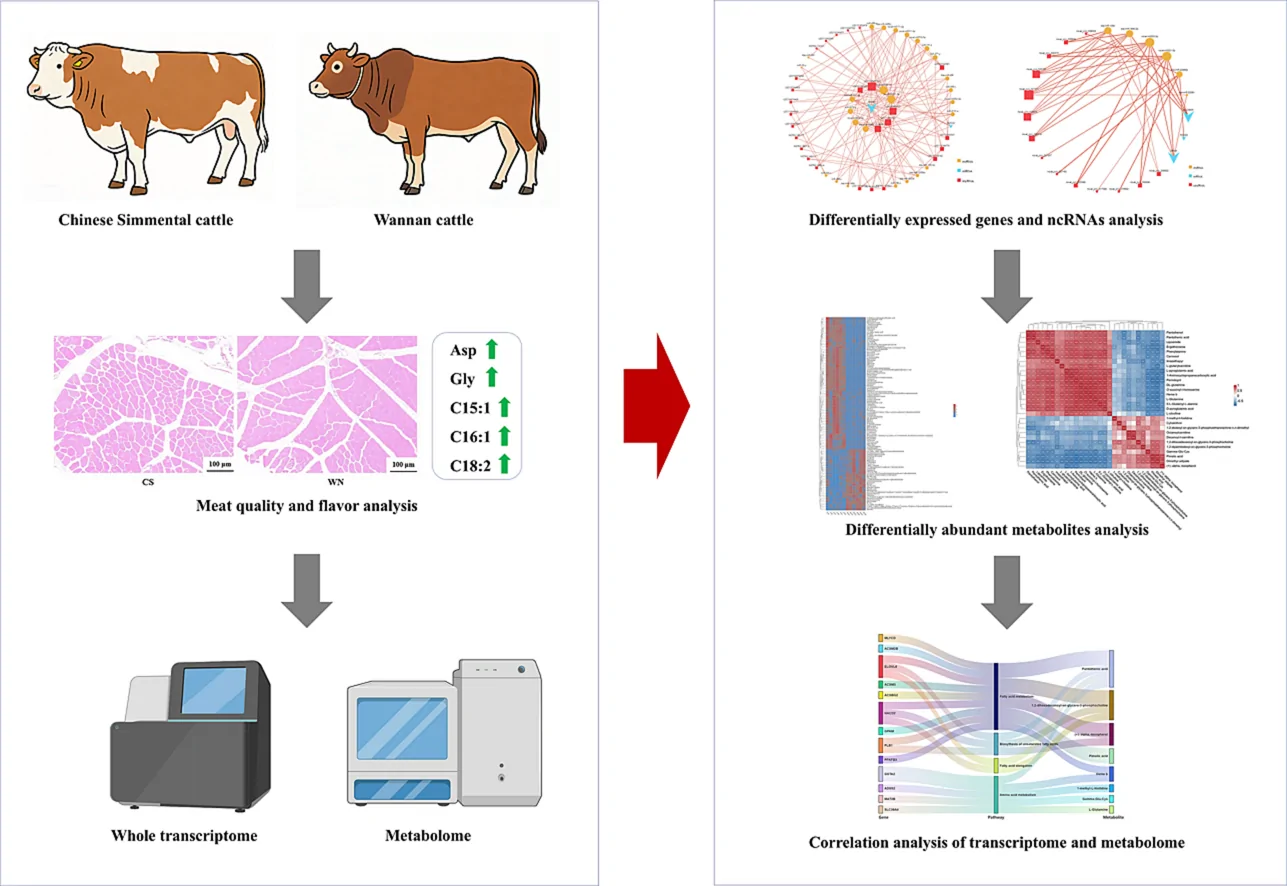

## 1 Introduction

Beef is recognized as an important source of high-quality protein, essential amino acids, and fatty acids in the human diet (McNeill & Van Elswyk, 2012). China, as one of the world's leading consumers and producers of beef, has witnessed a growing consumer preference for high-quality beef products, driven by rising living standards and health consciousness (Liu, Yang, et al., 2025).

Meat quality is a comprehensive trait that includes tenderness, water-holding capacity, intramuscular fat content, amino acid composition, fatty acid profile, and volatile flavor compounds (Khan et al., 2015). These attributes are closely associated with muscle fiber characteristics (Cho et al., 2024), metabolic pathways (Regmi et al., 2026), and gene expression patterns (Yu et al., 2023). In recent years, multi-omics approaches—including transcriptomics and metabolomics—have been widely used to explore the biological basis of meat quality in livestock such as yaks (Wu et al., 2022), sheep (Zhao et al., 2022), and pigs (Zhang et al., 2025). For instance, age-associated alterations in lipid molecules and flavor precursors significantly influence the sensory quality of yak meat (Wang et al., 2025). Similarly, muscle metabolic profiles in Sunit sheep across various ages have been elucidated through non-targeted and targeted metabolomics (Chen et al., 2025).

There are crucial differences in meat quality across different cattle breeds, which are influenced by genetics (Yu, Yang, et al., 2024), feeding management (Maughan et al., 2012), and slaughter age (Liu, Yu, et al., 2025). Local cattle breeds often exhibit better meat quality traits—such as tenderness, flavor, and nutritional value—compared to commercial crossbreeds (Zhao et al., 2025; Zhou et al., 2024). Chinese Simmental cattle (CS) are widely distributed throughout China and are characterized by rapid growth and high meat yield (Liu et al., 2020). In contrast, Wannan cattle (WN), a local breed native to southern Anhui Province, are known for their superior tenderness and unique flavor (Zhang et al., 2015).

Although previous studies compared meat quality across cattle breeds, most focused on conventional traits, with less attention paid to the underlying regulatory mechanisms integrated the transcriptional and metabolic levels. This study aimed to characterize the meat quality, differentially expressed genes (DEGs) and abundant metabolites (DAMs) of Chinese Simmental cattle and Wannan cattle, and to uncover the key metabolic pathways and regulatory networks underlying meat quality and flavor formation. The findings are expected to contribute to the genetic improvement of local cattle breeds and promote the development of high-quality beef production in China.

## 2 Materials and methods

### 2.1 Ethics statement

All methods were conducted in accordance with the relevant guidelines established by the Ministry of Agriculture of the People's Republic of China. All animal procedures were approved by the Ethics Committee of Anhui Agricultural University (SYXK 2021–009).

### 2.2 Animals and sample collection

Chinese Simmental cattle and Wannan cattle were raised in Zhenhua Cattle Breeding Family Farm in Jixi County, Xuancheng City, Anhui Province, China. A total of 12 unrelated bulls (Chinese Simmental, CS, *n* = 6; Wannan, WN, *n* = 6) were raised under uniform environmental and feeding conditions, adhering to China's beef cattle feeding standards. At the age of 36 months, all these 12 cattle (uncastrated males) were fasted for 24 h with free access to water, and slaughtered in the same batch. All samples of the *longissimus thoracis* (LT) muscle were harvested from each left half-carcass at the 13th rib height within 45 min post-mortem. For muscle fiber characterization, 2 cm^3^ pieces of LT muscle samples were cooled at 2 °C for 24 h, and then fixed in 4% paraformaldehyde. For whole transcriptomics and metabolomics, 1 cm^3^ pieces of LT muscle samples were snap-frozen in liquid nitrogen, stored at −80 °C, and subjected to omics analyses within 1 month. For amino acid and fatty acid composition measurement, LT muscle samples (100 g) were cooled at 2 °C for 24 h and then freeze-dried.

### 2.3 Measurement of muscle fiber characteristics

LT muscle samples (CS, *n* = 6; WN, *n* = 6) were fixed in 4% paraformaldehyde for 24 h, embedded in paraffin, sectioned into cross-sections, and stained with hematoxylin and eosin (H&E). Three random visual fields of fibers in each histological section were visualized using Caseviewer software. Three histological sections were used for muscle fiber measurement per animal. Muscle fiber diameter and the number of muscle fibers within fascicles were quantified via Image-Pro Plus software. The equivalent diameter of muscle fibers was calculated based on the cross-sectional area.

### 2.4 Measurement of amino acid composition

The freeze-dried LT samples (100 mg; CS, *n* = 6; WN, *n* = 6) were ground into fine powder, and added 10 mL 6 mol/L hydrochloric acid solution to a hydrolysis tube. The tube was purged with nitrogen, sealed hermetically, and then hydrolyzed at 110 °C for 22 h. Subsequently, 1 mL of the filtrate was transferred to a vacuum oven and dried at 50 °C. The dried residue was reconstituted in 2 mL sodium citrate buffer (pH 2.2), then filtered and transferred to an injection vial. The amino acids were measured with mixed amino acid standard solution as the external standard. Amino acids were analyzed using ninhydrin post-column derivatization ion-exchange chromatography (amino acid analyzer, LA8080, Hitachi, Japan) using a sulfonic acid-type cation exchange resin column (4.6 mm × 60 mm). The detection wavelength was 570 nm (440 nm for proline). Amino acid concentrations were calculated using an external standard method based on chromatographic peak area integration.

### 2.5 Measurement of fatty acid composition

The freeze-dried LT samples (5 g; CS, *n* = 6; WN, *n* = 6) were ground into fine powder, and supplemented with 2 mL of a C11:0 internal standard solution in methanol at a concentration of 5 mg/mL, 100 mg pyrogallic acid, 100 mg zeolite, 2 mL 95% ethanol, and 4 mL ddH_2_O. After adding 10 mL HCl, the mixture was hydrolyzed at 75 °C for 40 min. Following hydrolysis, 10 mL 95% ethanol and 50 mL diethyl ether-petroleum ether were added. The ether layer was evaporated to dryness, yielding the fat extract. For methyl-esterification, 8 mL 2% NaOH in methanol was added, followed by 7 mL 15% boron trifluoride in methanol and incubated at 80 °C. After cooling, 10 mL n-heptane and saturated NaCl was added, and the n-heptane layer (5 mL) was dehydrated with anhydrous sodium sulfate. The supernatant (fatty acid methyl esters) was measured with C11:0 as the internal standard. Fatty acid methyl esters were analyzed by gas chromatography (7890B, Agilent, CA, USA) using a highly polar biscyanopropyl siloxane stationary phase capillary column (100 m × 0.25 mm × 0.2 μm) with nitrogen carrier gas. The oven temperature program was as follows: 100 °C for 13 min; increased to 180 °C at 10 °C/min, held for 6 min; increased to 200 °C at 1 °C/min, held for 20 min; then increased to 230 °C at 4 °C/min, held for 10.5 min. The injection volume of samples was 1 μL and introduced in splitting mode (100:1) with an inlet temperature of 270 °C. Fatty acid concentrations were calculated using an internal standard method based on chromatographic peak area integration.

### 2.6 Whole transcriptome sequencing

Six samples of LT muscle (CS, *n* = 3; WN, *n* = 3) were selected from 12 cattle for whole transcriptome sequencing, matched by body weight. Total RNA was isolated and evaluated by an Agilent 2100 bioanalyzer and RNase-free agarose gel electrophoresis. The lncRNA and miRNA libraries were constructed on Illumina HiSeq 4000 and Novaseq6000 (Genedenovo, Guangzhou, China), respectively. Concurrently, Q30 and GC percentage of clean data were calculated for quality assessment. Clean reads were then mapped to the *Bos taurus* reference genome (ARS-UCD2.0) by HISAT2, and subsequent read assembly was performed with StringTie software.

### 2.7 Analysis of whole transcriptome sequencing

For lncRNA identification, transcripts with ≤2 exons or length ≤ 200 nt were excluded, along with those overlapping annotated lncRNAs. Novel lncRNAs were defined using CNCI (v2), CPC2 (0.9-r2), and Feelnc (Qiu et al., 2025). circRNAs were identified using CIRI2 software, requiring two back-spliced reads per candidate. Novel miRNAs were predicted using miRDeep2 based on genomic loci and hairpin structures.

DEGs, differentially expressed lncRNAs (DELs), circRNAs (DECs), and miRNAs (DEMs) between Chinese Simmental and Wannan cattle were discovered using edgeR (|log₂(fold change)| > 1 and adjusted *P* < 0.05). To predict trans-target DEGs of DELs, Pearson's correlation coefficients were set at adjusted *P* < 0.05 and |r| > 0.95. Gene Ontology (GO) analysis was employed using G:Profiler, and Kyoto Encyclopedia of Genes and Genomes (KEGG) analysis was applied using KOBAS.

Total RNA was recovered from LT muscle (CS, *n* = 3; WN, *n* = 3) corresponding to transcriptomic samples via the SPARKasy RNA Kit (AC0202, SparkJade, Jinan, China). cDNA was synthesized using TRUESCRIPT MASTMIX (PC7001, AidLab, Beijing, China). qRT-PCR was conducted on CFX96 (Bio-Rad, Hercules, CA, USA) using 2× Dual SYBR Green qPCR Mix (PC6201, AidLab). Ten DEGs were validated by qRT-PCR, and *GAPDH* served as the internal control (Supplementary Table S1).

Based on the competing endogenous RNA (ceRNA) hypothesis, lncRNA/circRNA–miRNA–mRNA interaction pairs were predicted using TargetScan based on the identified DEGs, DELs, DECs, and DEMs.

### 2.8 Untargeted metabolomics profiling

Untargeted metabolomics profiling was performed on 12 LT muscle samples (CS, *n* = 6; WN, *n* = 6) by Genedenovo Biotechnology Co., Ltd. Metabolite extraction was conducted following a standardized protocol involving the sequential procedures of grinding, ultrasonic treatment, incubation, centrifugation, lyophilization, reconstitution, and instrumental detection. The types and concentrations of metabolites were qualitatively and quantitatively evaluated using a UHPLC system (1290 Infinity LC, Agilent) coupled with an AB Triple TOF 6600 mass spectrometer (AB Sciex Pte. Ltd., Singapore) maintained at 40 °C*. mobile* phases consisted of water containing 25 mM ammonium acetate and 25 mM ammonium hydroxide (A) and acetonitrile (B). The mobile phases were delivered at a flow rate of 0.50 mL/min, and the injection volume was 2 μL. Mass spectrometry was performed in ESI (+/−) mode with the following parameters: source temperature 600 °C, sheath/aux gas 60/60 arb, and spray voltage ±5500 V. MS/MS spectra were acquired via information-dependent acquisition with declustering potential ±60 V, collision energy 35 ± 15 eV, dynamic exclusion of isotopes (4 Da window), and up to 10 MS/MS scans per cycle.

Peak extraction and alignment were acquired via MassLynx and Progenesis QI. Metabolite validation was implemented by matching against multiple databases, including METLIN database, accessible metabolome databases, an in-house constructed metabolite library, as well as theoretical fragment validation. After qualitative and quantitative analysis of metabolites, a series of downstream analyses were carried out. DAMs were discovered using |log_2_(fold change)| > 1, VIP ≥ 1, and *P* < 0.05 from independent Student's *t*-test, followed by functional enrichment analysis.

### 2.9 Integrative analysis of metabolome and transcriptome

To investigate the regulatory framework that may affect meat quality and flavor, the KEGG pathways that were co-enriched by all DAMs and DEGs were performed. The Spearman's correlation coefficients between DAMs and DEGs were calculated through pairwise comparison using the “cor” package in the R software. Subsequently, a potential regulatory network of key DAMs and DEGs closely associated with fatty acids and amino acids was constructed.

### 2.10 Statistical analysis

Data on muscle tissue characteristics, amino acid and fatty acid contents were analyzed using an independent-samples Student's *t*-test in SPSS 20.0 (IBM, NY, USA) and visualized using GraphPad Prism 9.0 (CA, USA). Statistical significance was displayed as ns (*P* > 0.05), *P < 0.05, and **P < 0.01. Relative gene expression was computed via the 2^−ΔΔCT^ method, and the gene expression levels of qRT-PCR and transcriptome were subjected to linear regression analysis using GraphPad Prism 9.0.

## 3 Results

### 3.1 Characteristics of muscle fibers

**Fig. 1:**
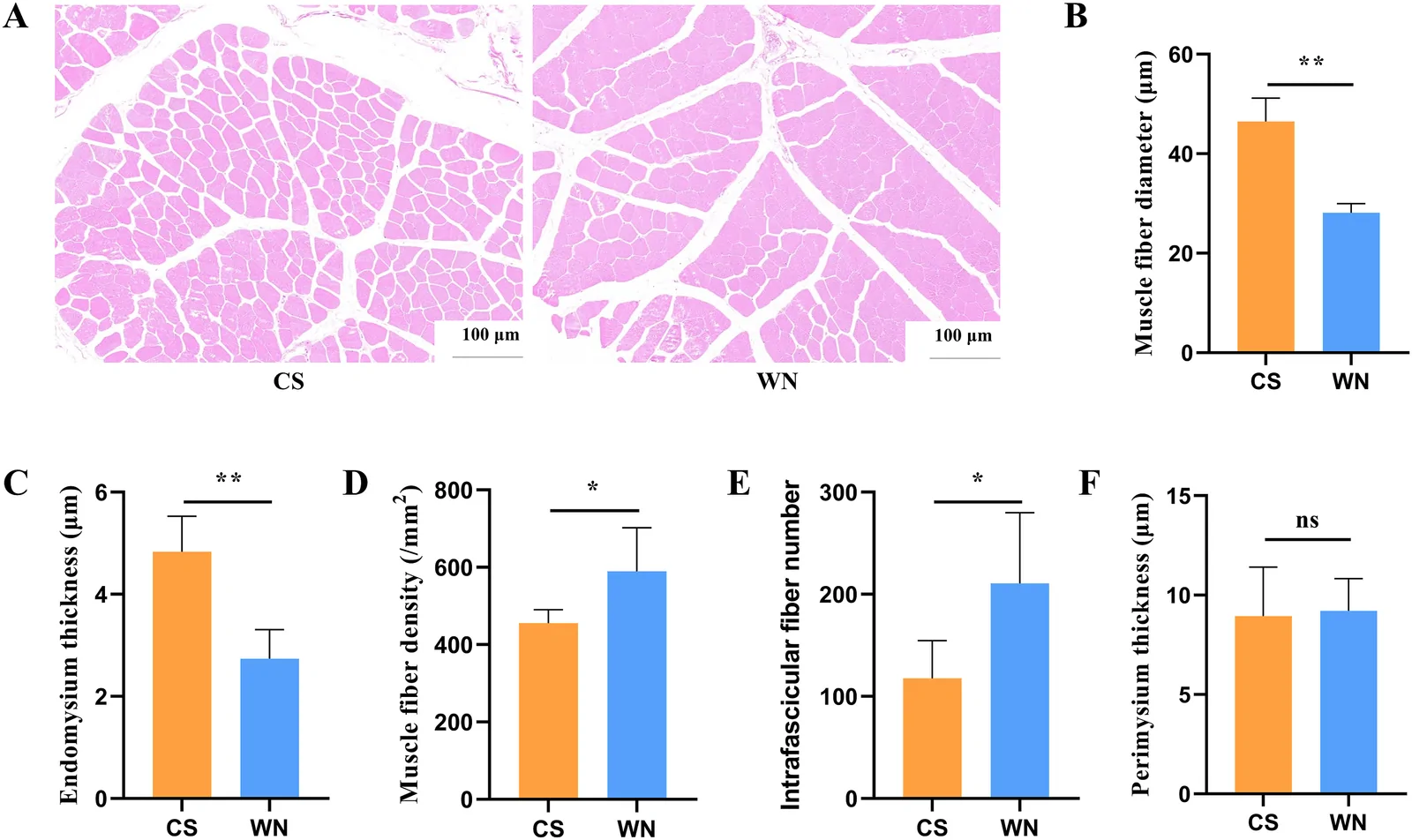
Muscle fiber characteristics in Chinese Simmental (CS) cattle and Wannan (WN) cattle. Hematoxylin-eosin (H&E) staining (A), muscle fiber diameter (B), endomysium thickness(C), muscle fiber density (D), intrafascicular fiber number (E), and perimysium thickness (F) of the *longissimus thoracis* (LT) muscle. Data are presented as mean ± SD. *P < 0.05 and **P < 0.01.

The muscle fiber diameter and endomysium thickness of WN cattle were significantly lower than those of CS cattle (Fig. 1A-1C, *P* < 0.01), while the muscle fiber density and intrafascicular fiber number of WN cattle were significantly higher than those of CS cattle (Fig. 1D and E, *P* < 0.05). No significant difference was observed in perimysium thickness between the two cattle breeds (Fig. 1F).

### 3.2 Composition of amino acids and fatty acids

Regarding the amino acid composition of the LT muscle (Table 1), no significant difference was observed in the total content of essential amino acids. Glutamic acid, aspartic acid, and lysine were the top three amino acids in both groups, contributing to beef flavor formation. More importantly, the contents of aspartic acid and glycine were markedly higher in WN cattle (*P* < 0.05).

**Table 1:** Meat amino acid composition (mg/100 g) of Chinese Simmental (CS) and Wannan (WN) cattle.

Trait	CS (*n* = 6)	WN (n = 6)	*P*-value
∑ Amino acids	19,424.00 ± 2886.36	20,855.09 ± 2353.02	0.104
∑ Essential amino acids	8070.61 ± 630.23	8269.67 ± 580.33	0.582
Threonine (Thr)^⁎^	1192.14 ± 138.70	1060.79 ± 219.70	0.244
Valine (Val)^⁎^	923.94 ± 145.60	1060.11 ± 91.94	0.081
Methionine (Met)^⁎^	488.44 ± 64.16^b^	612.85 ± 39.67^a^	0.002
Isoleucine (Ile)^⁎^	1003.99 ± 39.01^a^	876.11 ± 95.04^b^	0.012
Leucine (Leu)^⁎^	1630.60 ± 258.70	1749.02 ± 104.70	0.323
Phenylalanine (Phe)^⁎^	1126.17 ± 119.71	1009.95 ± 80.72	0.077
Lysine (Lys)^⁎^	1705.34 ± 366.68	1900.83 ± 204.72	0.288
∑ Non-essential amino acids	11,353.38 ± 1753.80^b^	12,585.42 ± 1516.53^a^	0.033
Aspartic acid (Asp)	1886.71 ± 153.90^b^	2115.21 ± 178.61^a^	0.039
Serine (Ser)	1032.39 ± 211.70	952.44 ± 161.30	0.479
Glutamic acid (Glu)	3245.38 ± 392.45	3560.84 ± 423.01	0.210
Glycine (Gly)	757.72 ± 123.95^b^	943.94 ± 110.96^a^	0.021
Alanine (Ala)	1170.89 ± 206.21	1369.11 ± 151.83	0.087
Cysteine (Cys)	149.11 ± 28.44^a^	94.49 ± 17.51^b^	0.002
Tyrosine (Tyr)	635.73 ± 173.30	703.62 ± 228.44	0.575
Histidine (His)	733.79 ± 149.79	852.61 ± 72.83	0.123
Arginine (Arg)	1213.22 ± 226.92	1385.45 ± 130.55	0.138
Proline (Pro)	528.45 ± 87.14	607.72 ± 41.49	0.072

Note: * represents essential amino acids. Data are presented as mean ± SD. Different letters represent significant differences (*P* < 0.05) between the two groups.

Among 28 fatty acids (Table 2), the contents of C15:1, C16:1, and C18:2n6c in WN cattle were markedly increased (*P* < 0.05), while the contents of C20:0, C22:0, C23:0, and C22:1 were markedly decreased (*P* < 0.05). Furthermore, the total MUFA, PUFA, UFA contents in WN cattle were markedly higher (*P* < 0.05), whereas no significant difference was noted in the total SFA content.

**Table 2:** Meat fatty acid composition (mg/100 g) of Chinese Simmental (CS) and Wannan (WN) cattle.

Trait	CS (*n* = 6)	WN (n = 6)	*P*-value
∑ SFA	2269.13 ± 333.05	2053.76 ± 266.60	0.244
C12:0	24.22 ± 4.28	23.07 ± 3.49	0.621
C13:0	5.80 ± 0.81	6.16 ± 0.92	0.502
C14:0	111.16 ± 22.18	104.91 ± 13.12	0.566
C15:0	10.05 ± 0.20	10.14 ± 0.14	0.354
C16:0	1278.48 ± 241.55	1171.95 ± 247.47	0.468
C17:0	15.32 ± 2.46	15.09 ± 1.94	0.862
C18:0	716.2 ± 161.97	633.22 ± 176.39	0.416
C20:0	24.71 ± 2.51^a^	17.95 ± 2.75^b^	0.001
C21:0	19.83 ± 2.06	19.25 ± 2.38	0.665
C22:0	11.84 ± 2.38^a^	8.86 ± 0.79^b^	0.026
C23:0	47.66 ± 7.23^a^	39.13 ± 3.23^b^	0.034
C24:0	3.86 ± 0.52	4.03 ± 0.49	0.589
∑ UFA	2805.37 ± 209.49^b^	3232.17 ± 316.82^a^	0.020
∑ MUFA	2550.35 ± 211.50^b^	2912.63 ± 300.99^a^	0.036
C14:1	14.03 ± 0.68	13.28 ± 2.06	0.421
C15:1	39.48 ± 3.32^b^	44.79 ± 3.99^a^	0.031
C16:1	194.03 ± 19.01^b^	366.83 ± 71.06^a^	0.000
C17:1	14.02 ± 1.59	15.13 ± 1.25	0.209
C18:1n9t	154.97 ± 29.82	131.76 ± 22.07	0.156
C18:1n9c	2114.87 ± 229.41	2323.39 ± 260.76	0.172
C20:1	8.36 ± 0.54	9.21 ± 1.14	0.129
C22:1	10.59 ± 1.37^a^	8.24 ± 1.61^b^	0.021
∑ PUFA	255.02 ± 37.09^b^	319.54 ± 31.02^a^	0.009
C18:2n6t	6.41 ± 0.75	6.57 ± 0.65	0.705
C18:2n6c	198.61 ± 39.09^b^	263.79 ± 29.66^a^	0.009
C18:3n3	9.08 ± 0.68	9.23 ± 0.49	0.663
C18:3n6	3.43 ± 0.45	3.39 ± 0.27	0.815
C20:2	6.30 ± 0.54	6.95 ± 1.25	0.267
C20:3n6	21.39 ± 2.69	18.66 ± 1.89	0.070
C20:4n6	4.76 ± 1.12	5.32 ± 0.47	0.285
C20:5n3	5.05 ± 0.73	5.63 ± 0.63	0.168

Note: SFA represents saturated fatty acid; MUFA represents monounsaturated fatty acids; PUFA represents polyunsaturated fatty acids; UFA represents unsaturated fatty acids. Data are presented as mean ± SD. Different letters represent significant differences (*P* < 0.05) between two groups.

### 3.3 Analysis of DEGs

**Fig. 2:**
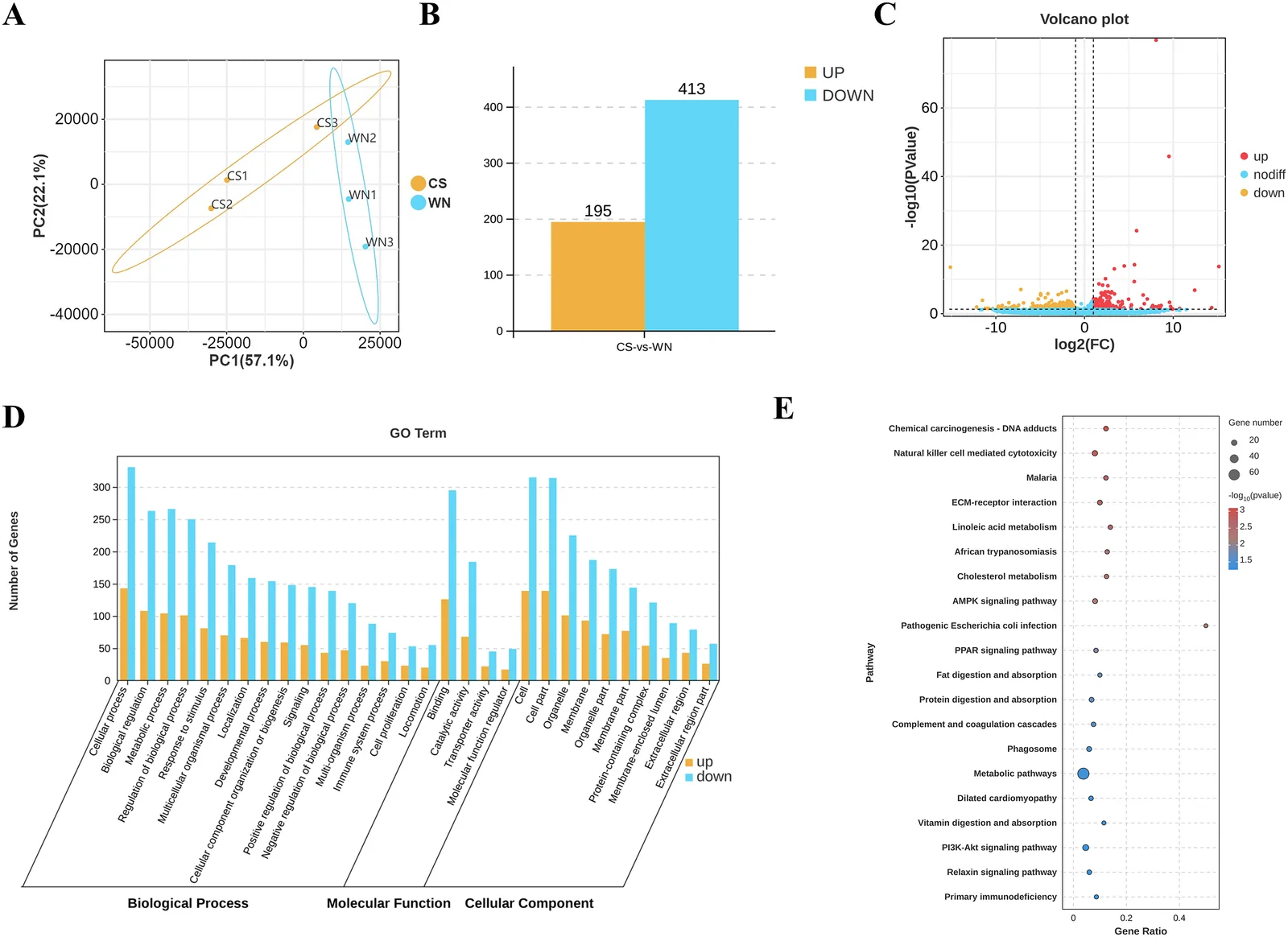
Differentially expressed genes (DEGs) between CS cattle and WN cattle. (A) Principal component analysis (PCA) of two groups. Column diagram (B), volcano plot (C), Gene Ontology (GO) enrichment analysis (D), and Encyclopedia of Genes and Genomes (KEGG) enrichment analysis (E) of DEGs. Up-regulated and down-regulated genes are defined as expression changes in WN cattle relative to CS cattle.

A total of 221,576,858 and 259,584,468 clean reads were obtained for CS and WN group, and the Q30 values ranged from 92.48% to 93.59% (Supplementary Table S2). The principal component analysis (PCA) result of mRNAs confirmed the good reproducibility of our transcriptome sequencing (Fig. 2A). The whole transcriptome sequencing data generated were submitted to NCBI database BioProject PRJNA1419575.

A total of 608 DEGs were identified, among which 195 were up-regulated and 413 were down-regulated (Fig. 2B and Supplementary Table S3). Among the DEGs associated with fatty acid or amino acid metabolism (Fig. 2C), *ACSM3*, *MLYCD*, *FADS6*, *PFKFB3*, *PLB1*, *MAT2B*, and *GSTA2* were upregulated, while *ELOVL6*, *HACD2*, *ACSM2B*, *ACSBG2*, *GPAM*, *ADSS2*, and *SLC36A4* were downregulated. GO enrichment analysis yielded 60 significantly enriched terms, and several terms were associated with muscle biology, including cellular process, biological regulation, metabolic process, catalytic activity, and transporter activity (Fig. 2D). KEGG analysis identified 271 pathways, with the top 20 enriched pathways including metabolic pathway, PI3K-Akt, AMPK, PPAR signaling pathway, linoleic acid metabolism, and cholesterol metabolism (Fig. 2E and Supplementary Table S4). Additionally, the expression dynamics obtained from qRT-PCR matched those from transcriptome sequencing, (Supplementary Fig. S1A). Regression analysis of the qRT-PCR and transcriptome sequencing showed a significant correlation with R^2^ = 0.9122 (*P* < 0.001) (Supplementary Fig. S1B), indicating the transcriptome data were high reliability.

### 3.4 Analysis of differentially expressed ncRNAs

**Fig. 3:**
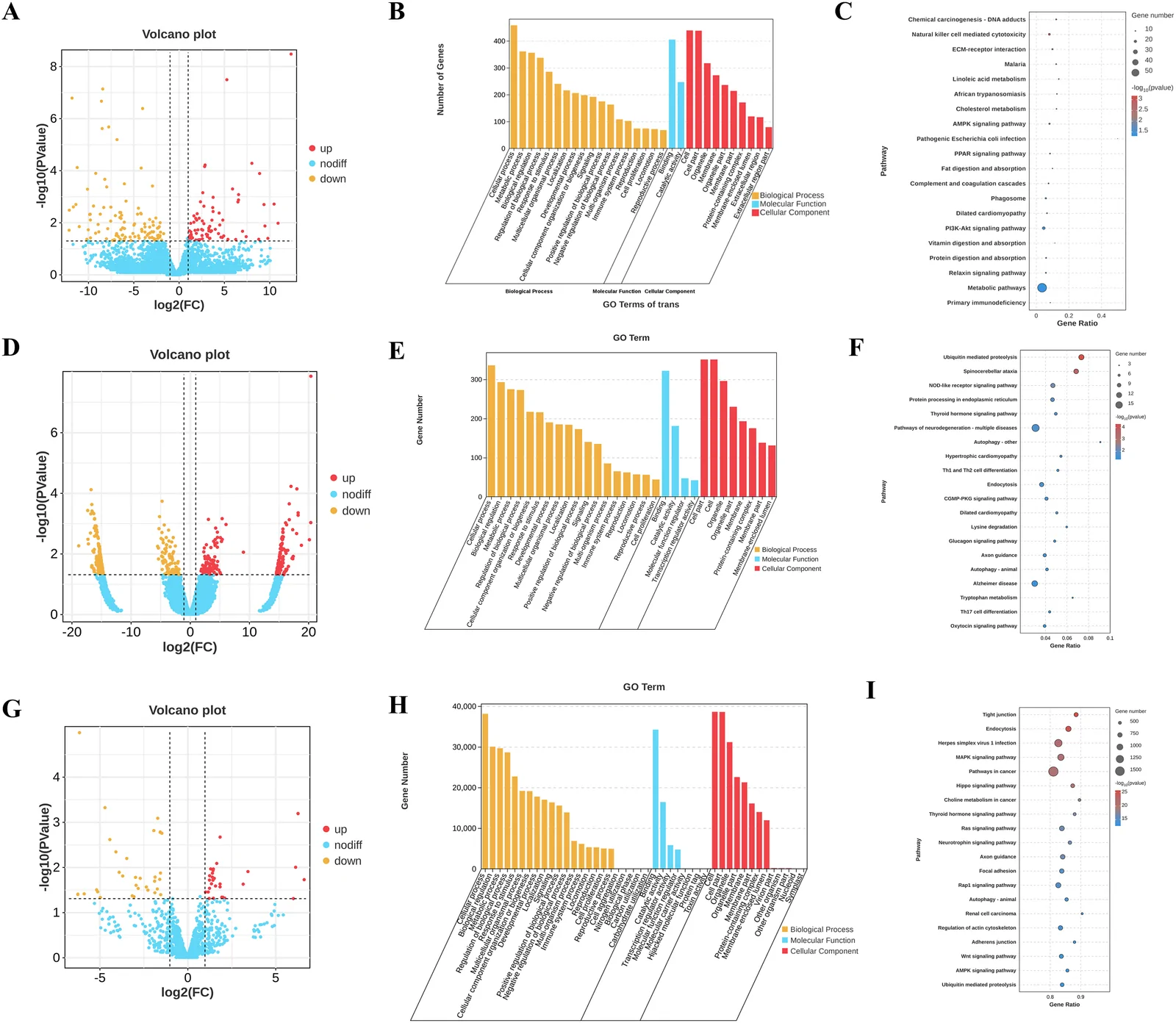
Differentially expressed non-coding RNAs between CS cattle and WN cattle. Volcano plot of differentially expressed lncRNAs (DELs) (A), circRNAs (DECs) (D), and miRNAs (DEMs) (G). GO enrichment analysis of trans-target genes of DELs (B), host genes of DECs (E), and target genes of DEMs (H). KEGG enrichment analysis of trans-target genes of DELs (C), host genes of DECs (F), and target genes of DEMs (I). Up-regulated and down-regulated non-coding RNAs are defined as expression changes in WN cattle relative to CS cattle.

A total of 7940 annotated and 719 novel lncRNAs were discovered from CS and WN cattle (Supplementary Fig. S2A and Supplementary Table S5). According to their genomic locations, these novel lncRNAs were classified into five categories (Supplementary Fig. S2B). The three most prevalent length ranges for lncRNAs were > 5000 nt, 500–1000 nt, and 1000–1500 nt (Supplementary Fig. S2C). Regarding exon number distribution, the most frequently observed exon counts for lncRNAs were 3, 2, and 4 (Supplementary Fig. S2D). A total of 190 DELs were discovered, comprising 78 up-regulated and 112 down-regulated transcripts (Fig. 3A and Supplementary Table S6). Co-expression analysis of DELs and their potential trans-target genes identified 6208 interaction pairs between 190 DELs and 591 DEGs (Supplementary Table S7). GO terms of trans-target genes included cellular process, biological regulation, metabolic process, and catalytic activity (Fig. 3B). Furthermore, pathways included linoleic acid metabolism, cholesterol metabolism, AMPK, PPAR, PI3K-Akt signaling pathway, and metabolic pathways (Fig. 3C and Supplementary Table S8).

A total of 39,999 circRNAs were discovered (Supplementary Table S9), among which those transcribed from exons accounted for the largest proportion (Supplementary Fig. S3A). CircRNAs with lengths ranging from 301 to 400 bp and > 3000 bp were the most abundant (Supplementary Fig. S3B). A total of 499 DECs were screened, comprising 228 up-regulated and 271 down-regulated transcripts (Fig. 3D and Supplementary Table S10). GO terms of host genes of DECs included cellular process, binding, and cell part (Fig. 3E). KEGG pathway included lysine degradation, cGMP-PKG, glucagon signaling pathway, and tryptophan metabolism (Fig. 3F and Supplementary Table S11).

A total of 1321 miRNAs with lengths ranging from 17 bp to 28 bp were identified, including 1100 known miRNAs and 221 novel miRNAs (Supplementary Fig. S3C, Supplementary Table S12). A total of 67 DEMs were detected, with 31 up-regulated and 36 down-regulated (Fig. 3G and Supplementary Table S13). GO terms of target genes of DEMs included cellular process, biological regulation, binding, cell part, and organelle (Fig. 3H). KEGG pathway included regulation of actin cytoskeleton, MAPK, Hippo, Wnt, and AMPK signaling pathway (Fig. 3I and Supplementary Table S14).

### 3.5 Construction of ceRNA network

**Fig. 4:**
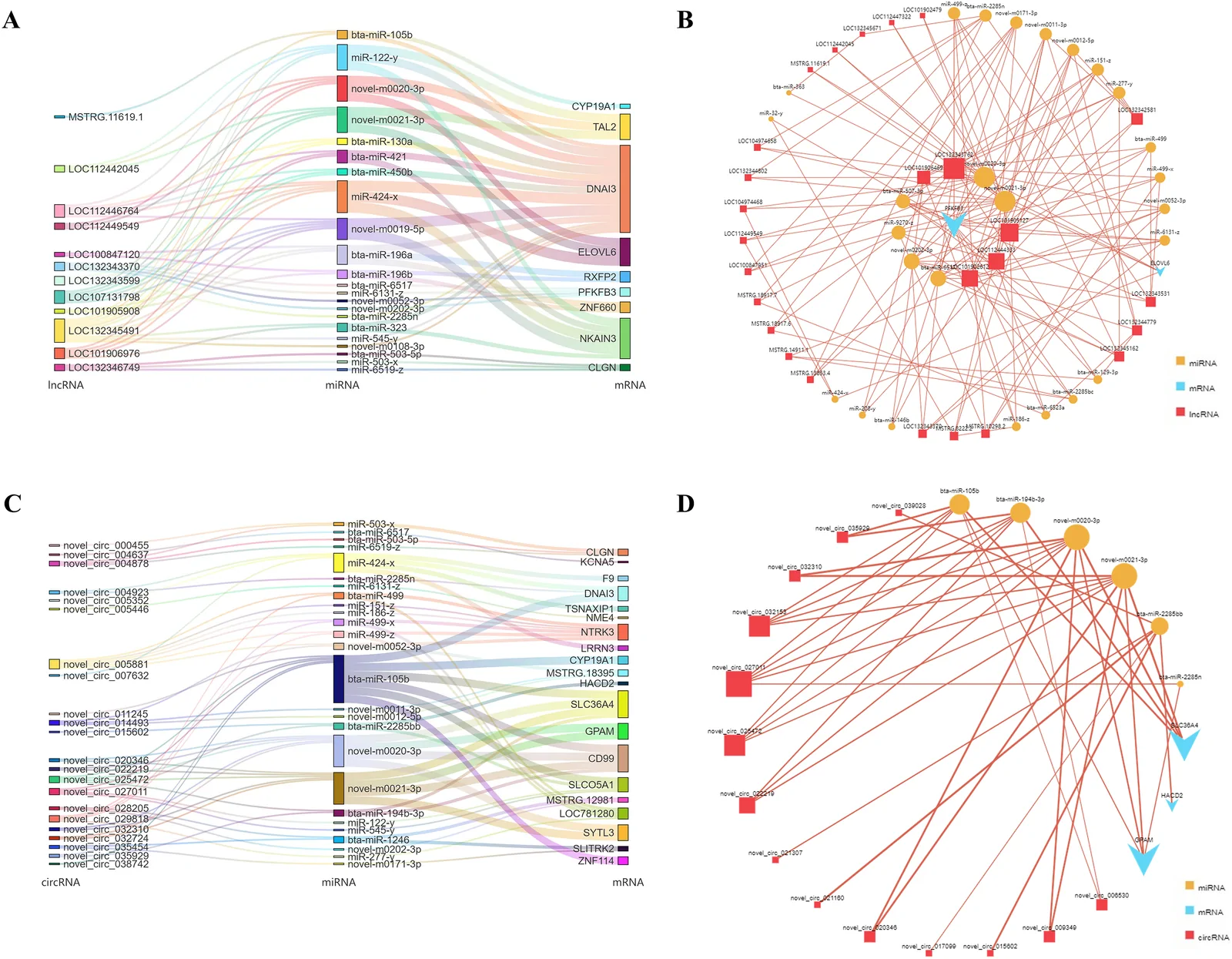
Competing endogenous RNA network in CS cattle and WN cattle. (A) Top 50 lncRNA-miRNA-mRNA interaction pairs. (B) lncRNA-miRNA-mRNA pairs interacting with key genes. (C) Top 50 circRNA-miRNA-mRNA interaction pairs. (D) circRNA-miRNA-mRNA pairs interacting with key genes.

A lncRNA-miRNA-mRNA axis analysis identified a total of 7757 ceRNA interaction pairs, encompassing 371 DEGs, 65 DEMs, and 159 DELs (Supplementary Table S15). As shown in Fig. 4A, the top 50 highly correlated lncRNA-miRNA-mRNA pairs contained *ELOVL6* and *PFKFB3*. Networks involving 26 DELs and 26 DEMs were constructed centered on *ELOVL6* and *PFKFB3* (Fig. 4B), represented by LOC112449549/miR-424/ELOVL6 and LOC132343370/miR-6517/PFKFB3 axes. Furthermore, a circRNA-miRNA-mRNA regulatory network analysis uncovered a total of 6186 ceRNA pairs, involving 432 DEGs, 64 DEMs, and 272 DECs (Supplementary Table S16). Fig. 4C showed that the top 50 highly correlated circRNA-miRNA-mRNA pairs included *HACD2*, *SLC36A4*, and *GPAM*. Additionally, networks encompassing 14 DECs and 6 DEMs were established around *HACD2*, *SLC36 A4*, and *GPAM* (Fig. 4D), represented by circ_015602/miR-2285/HACD2, circ_035929/miR-194-3p/SLC36 A4, and circ_022219/ m0020-3p/GPAM axes.

### 3.6 Analysis of DAMs and functional enrichment

**Fig. 5:**
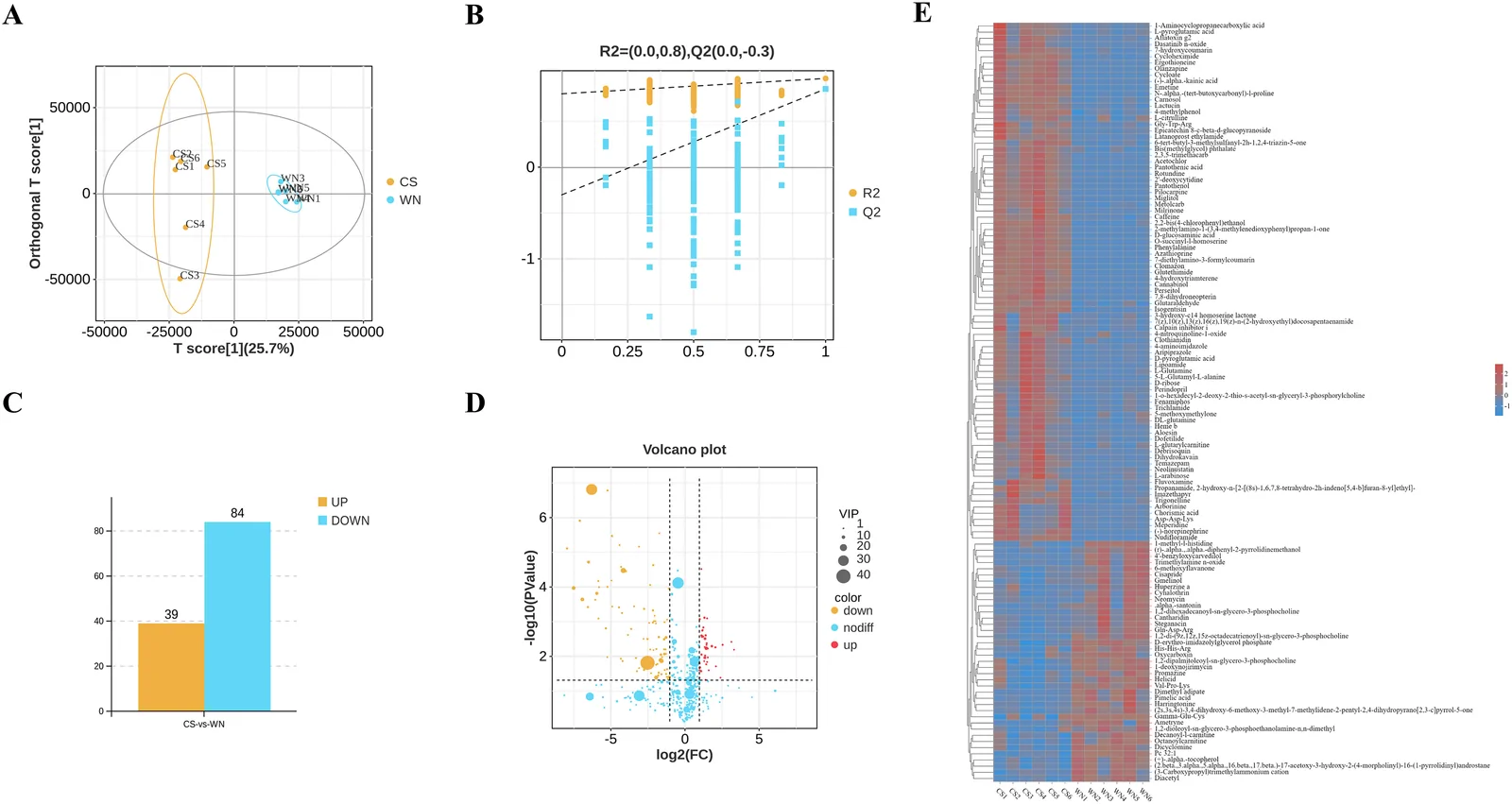
Identification and analysis of differentially abundant metabolites (DAMs) between CS cattle and WN cattle. (A) PCA of metabolites between two groups. (B) Orthogonal partial least squares-discriminant analysis (OPLS-DA) of two groups. Column diagram (C), volcano plot (D), and heatmap (E) of DAMs. Up-regulated and down-regulated metabolites are defined as abundant changes in WN cattle relative to CS cattle.

After metabolomic profiling and quantitative analysis, PCA and orthogonal partial least squares-discriminant analysis (OPLS-DA) validated an evident separation of samples (Fig. 5A and B). A total of 123 DAMs were identified, among which 39 metabolites were up-regulated and 84 were down-regulated (Fig. 5C-5E, and Supplementary Table S17). Compared with CS cattle, WN cattle exhibited higher contents of 1,2-dihexadecanoyl-sn-glycero-3-phosphocholine (16:0 PC, glycerophospholipid metabolism), 1,2-dipalmitoleoyl-sn-glycero-3-phosphocholine (16:1 (Δ9-Cis) PC, glycerophospholipid metabolism), 1,2-dioleoyl-sn-glycero-3-phosphoethanolamine-*N,N*-dimethyl (18:1 Dimethyl PE, glycerophospholipid metabolism), 1-methyl-L-histidine (histidine metabolism), gamma-Glu-Cys (glutathione metabolism), pimelic acid (biotin metabolism), and (+)-alpha-tocopherol (vitamin digestion and absorption), whereas the contents of pantothenic acid (pantothenate and CoA biosynthesis), lipoamide (lipoic acid metabolism), l-glutamine (glutamate metabolism), L-pyroglutamic acid (glutathione metabolism), and Heme b (ABC transporters) were significantly lower.

**Fig. 6:**
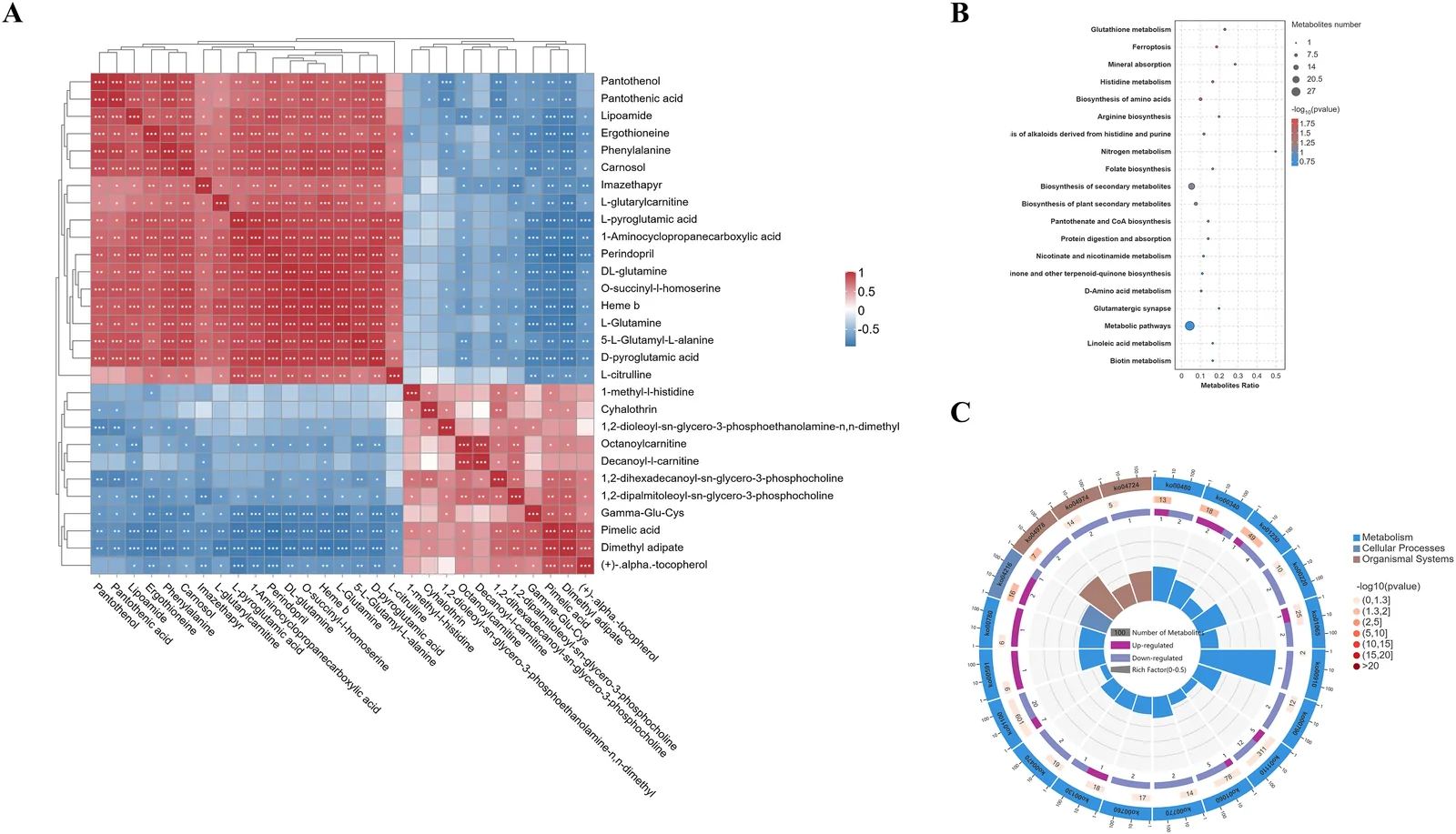
Correlation analysis and KEGG enrichment of DAMs. (A) Spearman's correlation analysis of DAMs. Bubble plot (B) and enrichment circle plot (C) of top 20 enrichment KEGG pathways of DAMs.

We performed correlation analysis on DAMs associated with fatty acid or amino acid metabolism (Fig. 6A). The results showed that 16:0 PC/16:1 (Δ9-Cis) PC was positively correlated with pimelic acid, (+)-alpha-tocopherol, and gamma-Glu-Cys, while negatively correlated with pantothenic acid, Heme b and l-glutamine; (+)-alpha-Tocopherol exhibited positive correlations with pimelic acid, gamma-Glu-Cys, and 1-methyl-L-histidine, and negative correlations with Heme b and l-glutamine; and Heme b was positively correlated with l-glutamine, while negatively correlated with pimelic acid and gamma-Glu-Cys. KEGG pathways included histidine metabolism, amino acid biosynthesis, arginine biosynthesis, D-amino acid metabolism, metabolic pathways, and linoleic acid metabolism (Fig. 6B). Correspondingly, in metabolic pathways, 7 were upregulated and 20 were downregulated; in biosynthesis of secondary metabolites, 5 were upregulated and 12 were downregulated; in biosynthesis of amino acids, 1 was upregulated and 4 were downregulated (Fig. 6C).

### 3.7 Integrated analysis of transcriptome and metabolome

**Fig. 7:**
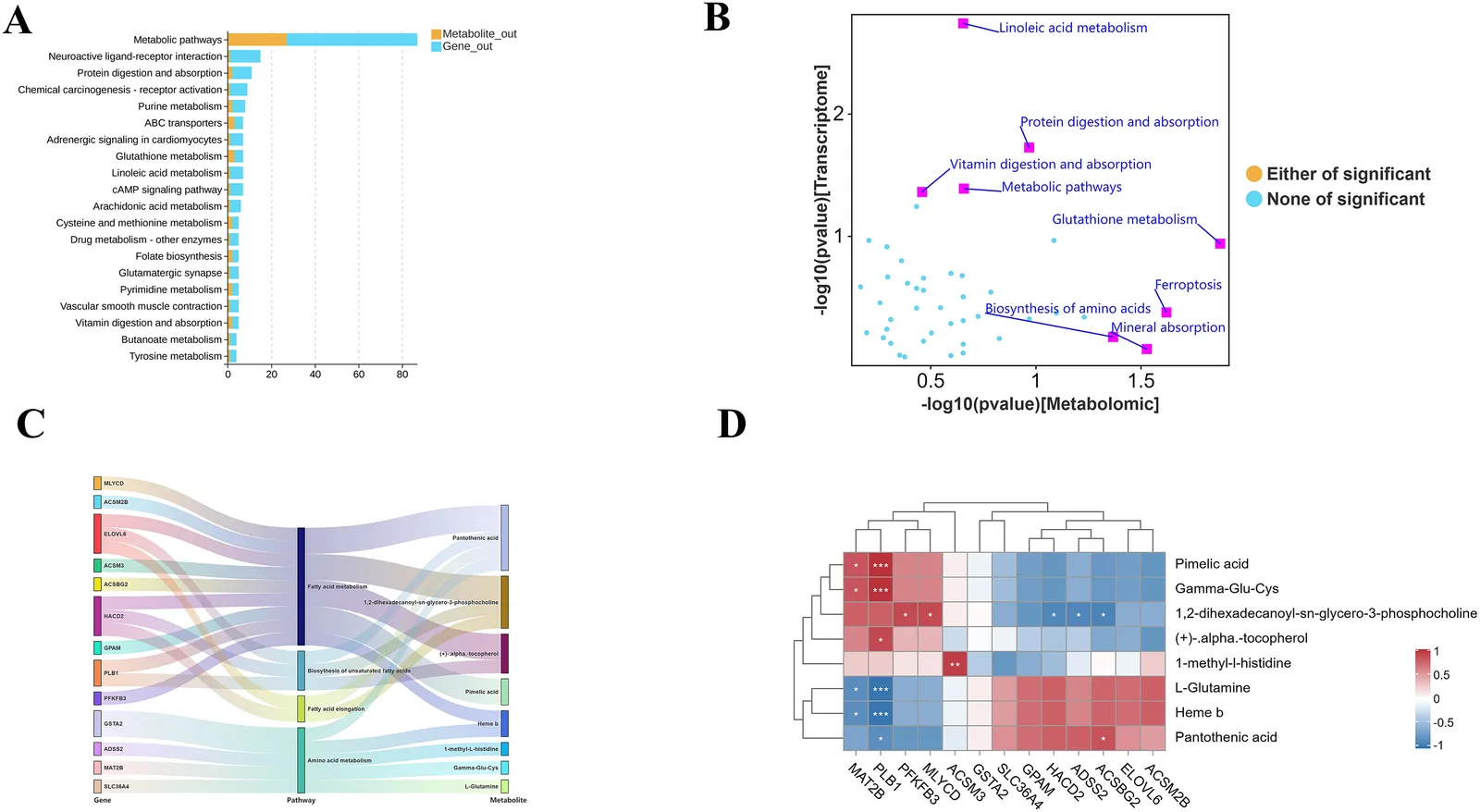
Integrated analysis of transcriptome and metabolome. Top 20 enrichment KEGG pathways (A) and significance pathway (B) analysis of DEGs and DAMs. Correlation network (C) and Spearman's correlation analysis (D) among DEGs and DAMs.

We discovered that 114 DEGs and 31 DAMs were co-enriched in 45 pathways. The top 20 pathways included metabolic pathways, linoleic acid metabolism, arachidonic acid metabolism, tyrosine metabolism, and cysteine and methionine metabolism (Fig. 7A and Supplementary Table S18). We identified significantly enriched pathways such as linoleic acid metabolism, protein digestion and absorption, metabolic pathways, vitamin digestion and absorption, glutathione metabolism, biosynthesis of amino acids (Fig. 7B). We focused on fatty acid metabolism, biosynthesis of unsaturated fatty acids, fatty acid elongation, and amino acid metabolism, and revealed these four pathways involved 13 DEGs and 8 DAMs (Fig. 7C). Collectively, key genes included *MLYCD*, *ACSM2B*, *ELOVL6*, *ACSM3*, *ACSBG2*, *HACD2*, *GPAM*, *PLB1*, *PFKFB3*, *SLC36A4*, *ADSS2*, *GSTA2*, and *MAT2B*, which were closely correlated with metabolites such as 16:0 PC, (+)-alpha-tocopherol, pantothenic acid, pimelic acid, Heme b, 1-methyl-L-histidine, gamma-Glu-Cys, and l-glutamine.

We performed correlation analysis on key DEGs and DAMs, and found that *PLB1* was positively correlated with pimelic acid, gamma-Glu-Cys, and (+)-alpha-tocopherol, while negatively correlated with l-glutamine, Heme b, and pantothenic acid; *MAT2B* was positively correlated with pimelic acid and gamma-Glu-Cys, but negatively correlated with l-glutamine and Heme b. 16:0 PC showed positive correlations with *PFKFB3* and *MLYCD*, and negative correlations with *HACD2*, *ADSS2*, and *ACSBG2* (Fig. 7D).

## 4 Discussion

Histological characteristics of muscle fibers serve as pivotal microscopic determinants of beef tenderness, juiciness, and flavor (Mo et al., 2023). Compared with CS cattle, WN cattle exhibit smaller muscle fiber diameter and endomysium thickness, coupled with higher muscle fiber density, thereby enhancing connective tissue encapsulation and improving tenderness and juiciness (Cho et al., 2024). Amino acids are the fundamental structural units of proteins, and skeletal muscles contain essential amino acids required for human nutrition (Skrlep et al., 2024). Further, umami−/sweet-related amino acids, including aspartic acid, glutamic acid, glycine, and alanine, are involved in the umami and sweet taste of meat (Lee et al., 2012). In this study, the contents of aspartic acid and glycine were higher in WN cattle than in CS cattle, suggesting that beef from WN cattle may have a more delicious flavor. The composition of fatty acids influences beef tenderness and flavor, and dietary intake of UFAs is beneficial to human health (Lee et al., 2019). MUFAs exhibit hypoglycemic and cholesterol-lowering effects, while PUFAs play pivotal roles in enhancing human immunity, blood circulation, and cardiovascular health (Rahman et al., 2023). In both breeds of our study, C18:1n9c was the dominant fatty acid, contributing to superior flavor and juiciness. WN cattle exhibited higher total UFA content compared to Chinese Simmental cattle, with elevated levels of C15:1, C16:1, and C18:2n6c. Notably, UFAs are critical regulators of muscle fiber diameter, which may underlie the smaller fiber diameter and higher fiber density observed in WN cattle (Jeong et al., 2017).

Whole transcriptome enabled the identification of DEGs, differentially expressed ncRNAs and ceRNA regulatory networks. FADS6 catalyzes introduction of double bonds into C16 and C18 fatty acids, serving as a critical enzyme for UFA biosynthesis and enhancing fatty acid unsaturation (Cui et al., 2020). PLB1 is involved in the hydrolysis of phospholipids, and PLB1 knockout reduces the production of free UFAs (Ferreira et al., 2018). Conversely, ELOVL6 and HACD2 function as elongases for long-chain fatty acids (Du et al., 2022; Zappaterra et al., 2018). PFKFB3 regulates AMPK pathway activity by promoting glycolysis to generate key substrates for fatty acid synthesis, namely acetyl-CoA and glycerol-3-phosphate (Griesel et al., 2021). In this study, *FADS6, PLB1,* and *PFKFB3* were highly expressed in WN cattle, whereas *ELOVL6* and *HACD2* showed lower expression, which may contribute to high abundances of UFAs. GSTA2 preserves the structural integrity and functional properties of umami−/sweet-related amino acids (Yang et al., 2001). ADSS2 catalyzes the adenylation of inosine monophosphate with aspartic acid, activating the AMPK pathway to regulate amino acid uptake and utilization (Li et al., 2007). SLC36A4 mediates neutral amino acid transport, exhibiting a high binding affinity for proline and tryptophan but a low affinity for glycine and alanine (Fan et al., 2016). In the present study, *GSTA2* was highly expressed in WN cattle, while *ADSS2* and *SLC36A4* showed low expression levels, which may contribute to high abundances of aspartic acid and glycine.

Comprehensive analysis of DEGs and differentially expressed ncRNAs revealed that PI3K-Akt, AMPK, and PPAR signaling pathways play pivotal roles in fatty acid and amino acid metabolism. PI3K-Akt pathway promotes fatty acid synthesis by activating SREBP-1c and enhances amino acid uptake via activating mTOR complex(Gu et al., 2025). AMPK inhibits fatty acid synthesis through phosphorylation of acetyl-CoA carboxylase while promoting fatty acid oxidation, and it regulates amino acid metabolism by suppressing mTOR activity (Woonnoi et al., 2023). PPAR pathway regulates fatty acid synthesis, β-oxidation, and lipid storage, and it can also influence the activity of the AMPK pathway (Wang et al., 2026). Through ceRNA network analysis, we identified DEGs and DEMs associated with fatty acid and amino acid metabolism. Notably, ELOVL6 was a potential target of miR-363 and miR-424, while SLC36A4 was targeted by miR-194. miR-363 upregulates SREBP-1c and its downstream factors associated with lipid synthesis (Wang et al., 2024). miR-424 inhibits lipid droplet accumulation via repressing both lipid anabolic and catabolic pathways (Li et al., 2026). miR-194 modulates intracellular amino acid homeostasis by targeting SLC36A4 (Liu et al., 2024). In the present study, although we constructed the ceRNA network, its underlying regulatory mechanisms remain to be further validated in subsequent research.

Metabolomics provides terminal and functional direct evidence for elucidating meat quality and flavor formation (Liu et al., 2025). Upon binding of 16:0 PC to C16:0, the esterification and storage of C18:2n6c are promoted through the catalysis of phospholipase and lysophosphatidylcholine acyltransferase (Shindou & Shimizu, 2009). 16:1 (Δ9-Cis) PC and 18:1 Dimethyl PE, glycerophospholipids containing C16:1 and C18:1 fatty acid chains, respectively, activate phospholipid biosynthesis pathways to enhance MUFA synthesis or uptake (Frahm et al., 2013). (+)-alpha-Tocopherol scavenges lipid-soluble reactive oxygen species and blocks lipid peroxidation chain reactions, thereby inhibiting oxidative stress-induced fatty acid degradation (Jerzykiewicz et al., 2013). Pimelic acid, an intermediate product of β-oxidation for C15:0 and C17:0, promotes saturated fatty acid metabolism and lipid deposition (Cronan & Lin, 2011). Lipoamide facilitates carnitine-mediated transport of acyl-CoA into mitochondria, upregulating fatty acid β-oxidation and lipolysis while suppressing intramuscular triglyceride accumulation (Solmonson & DeBerardinis, 2018). 1-Methyl-L-histidine promotes the release of umami−/sweet-related amino acids, and its expression correlates negatively with muscle fiber diameter, serving as a biomarker for meat tenderness (Wu, 2020). Gamma-Glu-Cys enhances antioxidant capacity and mitigates reactive oxygen species-mediated oxidative damage to umami−/sweet-related amino acids (Broadhead et al., 2011). High expression levels of 16:0 PC, 16:1 (Δ9-Cis) PC, 18:1 Dimethyl PE, (+)-alpha-tocopherol and pimelic acid in WN cattle may contribute to elevated UFA content, whereas elevated 1-methyl-L-histidine and gamma-Glu-Cys may be linked to increased aspartic acid and glycine accumulation.

Integrated transcriptomic and metabolomic analysis serves as a pivotal technological strategy in meat quality research, bridging gene expression regulation with terminal metabolites. Based on the analysis of intramuscular fat and meat color in Enshi black pigs, niacin and nicotinamide metabolism was revealed (Zhan et al., 2022). Flavor formation mechanisms in Chalu black pigs were elucidated through identification of amino acid metabolism, glycolysis/gluconeogenesis, and cellular aromatic compound (Zhang et al., 2025). Meat quality analysis of Qinchuan cattle identified *ACOX3*, *HACD2*, *SCD5*, and erucic acid, purine metabolism, pyrimidine metabolism, and amino acid metabolism (Yu et al., 2023; Yu, Yu, et al., 2024). Meat quality and flavor analysis of Liaoyu white cattle highlighted glycolysis, TCA cycle and fatty acid metabolism (Tan et al., 2025). Analysis of intramuscular fat deposition in Jiaxian Red cattle discovered the alpha-linolenic acid- β-oxidation/l-carnitine-myeloperoxidase regulatory axis (Zhang et al., 2026). In this study, we focused on fatty acid and amino acid metabolism, identifying *ELOVL6*, *HACD2*, *PLB1*, *ADSS2*, *GSTA2*, and 16:0 PC, (+)-alpha-tocopherol, pantothenic acid, l-glutamine, gamma-Glu-Cys, 1-methyl-L-histidine. Nevertheless, limitations of this study include the unclear mechanisms of key DEGs and the lack of experimental validation for the ceRNA regulatory axes. Future work will involve functional validation of key candidate genes and ceRNA networks in vivo or in vitro to uncover the molecular basis of breed-specific meat quality differences.

## 5 Conclusion

This study investigated the differences in muscle fiber characteristics, amino acid and fatty acid composition between Wannan cattle and Chinese Simmental cattle. Whole-transcriptome and metabolome analyses uncovered key genes and metabolites involved in fatty acid metabolism (*FADS6*, *PLB1,* 16:0 PC, 16:1 (Δ9-Cis) PC, 18:1 Dimethyl PE) and amino acid metabolism (*GSTA2*, *ADSS2*, gamma-Glu-Cys, 1-methyl-L-histidine, l-glutamine). However, this study only identified key genes through multi-omics analysis, and further experimental validation is required to elucidate the molecular mechanisms by which these genes affect beef quality. Overall, our findings contribute to an understanding of formation of meat quality and flavor, and provide insights for beef quality improvement.

## CRediT authorship contribution statement

**Ning Song:** Writing – original draft, Methodology, Investigation, Formal analysis, Data curation. **Jia Wang:** Visualization, Formal analysis, Data curation. **Xiang Meng:** Visualization, Formal analysis. **Shuangshuang Cui:** Validation, Investigation. **Sihua Jin:** Supervision, Resources. **Hang Tang:** Investigation, Data curation. **Yunhai Zhang:** Resources, Project administration. **Hongyu Liu:** Writing – review & editing, Supervision, Project administration, Conceptualization.

## Ethics statement

All methods were conducted in accordance with the relevant guidelines established by the Ministry of Agriculture of the People's Republic of China. All animal procedures were approved by the Ethics Committee of Anhui Agricultural University (SYXK 2021–009).

## Funding

This work was funded by the Project of Beef Cattle Revitalization of Anhui Provincial Science and Technology Department (202513b10050014, 202513b10050019).

## Declaration of competing interest

The authors declare that they have no known competing financial interests or personal relationships that could have appeared to influence the work reported in this paper.

## Data Availability

Data will be made available on request.
