# Supplementary material for: Breed-specific meat quality and flavor characteristics of Wannan and Chinese Simmental cattle revealed by whole transcriptome and metabolome analysis
Source: Food Chem (Oxf). 2026 Sep 1;13:100451. doi: 10.1016/j.fochms.2026.100451 (PMC13572015; doi:10.1016/j.fochms.2026.100451)
Supplement: Supplementary file 1 [file mmc1.docx]

**Supplementary Fig. S1.** Validation of DEGs by qRT-PCR. (A) Comparison between qRT-PCR and transcriptome measurements of gene expression levels of 10 DEGs. (B) Regression analysis of log2(WN/CS) ratios between qRT-PCR and transcriptome.

**Supplementary Fig. S2.** Characterization of lncRNAs in CS cattle and WN cattle. (A) Venn diagram of coding potential analysis by CPC2, CNCI and Feelnc. (B) Classification of novel lncRNAs. (C) The length distribution of lncRNAs and mRNAs. (D) The exon number distribution of lncRNAs and mRNAs.

**Supplementary Fig. S3.** Characterization of circRNAs and miRNAs in CS cattle and WN cattle. (A) Distribution of circRNAs in the genome. (B) Length distribution of circRNAs. (C) Length distribution of miRNAs.
